# *In silico* design of a promiscuous chimeric multi-epitope vaccine against *Mycobacterium tuberculosis*

**DOI:** 10.1016/j.csbj.2023.01.019

**Published:** 2023-01-16

**Authors:** Binda T. Andongma, Yazheng Huang, Fang Chen, Qing Tang, Min Yang, Shan-Ho Chou, Xinfeng Li, Jin He

**Affiliations:** a State Key Laboratory of Agricultural Microbiology & Hubei Hongshan Laboratory, College of Life Science and Technology, Huazhong Agricultural University, Wuhan, Hubei 430070, PR China; b Key Laboratory of Molecular Biophysics of the Ministry of Education, College of Life Science and Technology, Huazhong University of Science and Technology, Wuhan, Hubei 430070, PR China; c CAS Key Laboratory of Special Pathogens and Biosafety, Center for Biosafety Mega-Science, Wuhan Institute of Virology, Chinese Academy of Sciences, Wuhan 430071, PR China

**Keywords:** Tuberculosis, *Mycobacterium tuberculosis*, Multi-epitope vaccine, Antigenicity, Immunogenicity, Immuno-informatics, Immune cell receptors, Adjuvant

## Abstract

Tuberculosis (TB) is a global health threat, killing approximately 1.5 million people each year. The eradication of *Mycobacterium tuberculosis*, the main causative agent of TB, is increasingly challenging due to the emergence of extensive drug-resistant strains. Vaccination is considered an effective way to protect the host from pathogens, but the only clinically approved TB vaccine, Bacillus Calmette-Guérin (BCG), has limited protection in adults. Multi-epitope vaccines have been found to enhance immunity to diseases by selectively combining epitopes from several candidate proteins. This study aimed to design a multi-epitope vaccine against TB using an immuno-informatics approach. Through functional enrichment, we identified eight proteins secreted by *M. tuberculosis* that are either required for pathogenesis, secreted into extracellular space, or both. We then analyzed the epitopes of these proteins and selected 16 helper T lymphocyte epitopes with interferon-γ inducing activity, 15 cytotoxic T lymphocyte epitopes, and 10 linear B-cell epitopes, and conjugated them with adjuvant and Pan HLA DR-binding epitope (PADRE) using appropriate linkers. Moreover, we predicted the tertiary structure of this vaccine, its potential interaction with Toll-Like Receptor-4 (TLR4), and the immune response it might elicit. The results showed that this vaccine had a strong affinity for TLR4, which could significantly stimulate CD4^+^ and CD8^+^ cells to secrete immune factors and B lymphocytes to secrete immunoglobulins, so as to obtain good humoral and cellular immunity. Overall, this multi-epitope protein was predicted to be stable, safe, highly antigenic, and highly immunogenic, which has the potential to serve as a global vaccine against TB.

## Graphical Abstract

**Figure:**
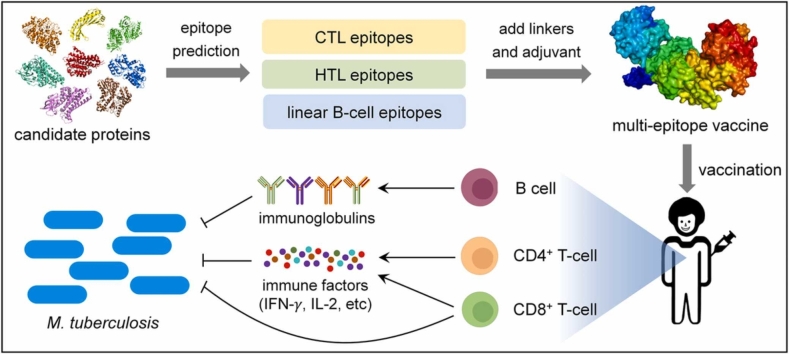

## 1 Introduction

Tuberculosis (TB), a highly contagious disease caused by *Mycobacterium tuberculosis*, is ranked by World Health Organization (WHO) as the top cause of death from a single infectious agent [1], [2], [3]. In 2021, the estimated number of TB deaths and new cases reached 1.6 million and 10.6 million, respectively [4]. Currently, the clinical treatment of TB is relatively scarce, and the combination of multiple antimicrobial drugs is mainly used. This chemotherapy cycle is very long, usually taking nine to twelve months, or even longer [5], which increases the risk of drug-resistant mutations in *M. tuberculosis* [6], [7]. In recent years, chemotherapy has become less effective because of the emergence and increasing proportion of multi-drug and extensively drug-resistant *M. tuberculosis* [6]. Preventing TB from developing may be more effective than treating it. Vaccination is well known to be an effective way to protect the host from pathogenic bacteria [8]. Currently, Bacillus Calmette-Guérin (BCG), developed over 100 years ago, is the only clinically approved TB vaccine [9]. Unfortunately, BCG only protects newborns and infants and is largely ineffective against adolescents and adults [2], [10], although WHO reports that 89% of TB cases in 2021 were adults [4]. Therefore, there is an urgent need to develop a novel and effective anti-TB vaccine, especially for adolescents and adults.

TB vaccine development is complicated by multiple features of mycobacteria, such as latent infection, persistence, and immune evasion [11], [12], [13]. An ideal TB vaccine should be designed to target the proteins/pathways responsible for these properties in *M. tuberculosis* and be able to efficiently induce CD4^+^ and CD8^+^ T cell-mediated immune responses [14]. Moreover, an effective vaccine should also target the host's major histocompatibility complexes (MHC), which are highly polymorphic [15]. These characteristics put forward very high requirements for the versatility of the vaccine, which obviously cannot be achieved by a single natural protein. Multi-epitope vaccine, a recombinant protein consisting of a series of or overlapping epitopes (peptides) [16], is a novel type of vaccine candidate that may address the above issues. In recent years, multi-epitope vaccines have attracted much attention due to their advantages of higher immunity and lower allergenicity than conventional vaccines [17], [18]. Currently, multi-epitope vaccines have been designed against many pathogenic microorganisms, including *Shigella* spp. [19], foot-and-mouth disease virus [20], *Helicobacter pylori* [21], [22], hepatitis B virus [23], *Toxoplasma gondii* [24], *Leishmania infantum* [25], Nipah virus [26], *Onchocerca volvulus* [27], *Pseudomonas aeruginosa* [28], and leukosis virus [29]. In particular, the emergence of the COVID-19 pandemic has strengthened the application of this technology [16], [30], [31], [32]. As for TB, several multi-epitope vaccines have been designed to target inherently active TB [33], [34], [35], [36], [37], [38], [39] and latent TB [40], [41]. Among them, three vaccine candidates were designed in the form of DNA [34], [36], [40], and two of them incorporated epitopes into the protein backbones to generate recombinant vaccines [34], [36]. It should be noted that the candidate proteins for some of the above multi-epitope vaccines are randomly selected, and the population coverage of these vaccines requires further studies. Moreover, two multi-epitope TB vaccine candidates with broad population coverage were designed, one epitope selected from immunogenic exosomes vesicle proteins with pathogenic properties [39], the other does not focus on candidate proteins, but directly selects highly conserved and experimentally validated epitopes from the Immune Epitope Database (IEDB) [38]. However, these candidate proteins lack functional enrichment, and the ability of vaccine candidates to induce interferon-γ (IFN-γ) secretion remains to be improved.

Previous study has deduced that rational optimization of epitopes can be achieved by a combination of MHC binding capacity and the epitope’s ability to react with T cell receptors [42]. Furthermore, they predicted that vaccines with cytotoxic T lymphocyte (CTL) A1, A2, A3, A24, and B7 binding epitopes would have coverage of nearly 100% in the major ethnic groups (Blacks, Asians, Hispanics, and Caucasians). However, until now there has been no similar approach to design a TB vaccine. In this study, we designed a highly promiscuous multi-epitope TB vaccine using various antigenic features of eight function-enriched proteins. The chimeric vaccine candidate possesses 15 CTL epitopes, 16 helper T lymphocyte (HTL) epitopes with IFN-γ-inducing properties, and 10 linear B-cells epitopes. Immuno-informatics analysis demonstrated that this vaccine candidate was ‘all-encompassing’, making it a potential cornerstone to achieve the ‘The End TB strategy’.

## 2 Materials and method

### 2.1 Protein selection and sequence retrieval

To construct a multi-epitope vaccine against TB, we first selected proteins of the *M. tuberculosis* complex, which are deposited in the IEDB database [43] and have been validated as MHC class I and II binding epitopes. Amino acid sequences (primary structure) of proteins from *M. tuberculosis* H37Rv strain were obtained from the UniProt database [44]. Alignment-independent predictions of prospective antigens based on physicochemical properties were obtained from the VaxiJen 2.0 server [45], which underwent automatic and cross-covariance (ACC) transformation of protein sequences into a uniform vector of major amino acid properties, with the antigenicity threshold set at 0.4 for each bacterial protein [45], [46]. Functional annotation of proteins was assessed using Database for Annotation, Visualization and Integrated Discovery (DAVID) 6.8 [47]. Secreted proteins were further enriched using two categories: extracellular space and pathogenesis through the DAVID and BioCyc [48] databases, respectively. The proteome of *Homo sapiens* GRCh38.p13 was downloaded in FASTA format from the National Centre for Biotechnology Information (NCBI) database [49]. BLASTp was used to predict homology (E-value =1e-5) between secreted proteins and *H. sapiens* proteins.

### 2.2 T-cell epitope prediction

Prediction and selection of epitopes are crucial steps in the construction of multi-epitope vaccines. MHC I molecules bind short peptides (9–11 amino acids) because the peptide-binding cleft of MHC I molecules consisting of a single α chain is closed [50]. The freely accessible NetMHCpan-4.1 [51] was used for CTL epitopes prediction, which uses NNAlign_MA to generates percentage ranks (% rank) based on a combination of MHC I binding affinities and eluted ligands. The "% Ranking" of a query sequence was determined by comparing its prediction score to the distribution of prediction scores for the relevant MHC calculated using a set of randomly chosen native peptides. Epitopes with a % ranking < 0.5% were considered strong binders, while epitopes with a % ranking < 2% were considered weak binders [51]. Although up to 12 supertype MHC class I epitopes can be predicted on the server, we only used A1, A2, A3, A24, and B7 because these five supertypes basically cover 100% of the major human races [42]. We selected strong binders and predicted their antigenicity using VaxiJen2.0 [45], then, we predicted class I immunogenicity using the International Epitope Database (IEDB) [52], which uses 3-fold cross validation. Finally, we arranged epitopes that were both antigenic and immunogenic according to % ranking and selected 15 low-scoring epitopes, three for each supertype and at least one for each candidate protein, except for candidate protein that could not have a strong CTL binding epitope that are antigenic and immunogenic. Finally, IC50 values for each CTL epitope were predicted from NetMHC-4.0 [53].

Class II MHC molecule bind to antigenic peptides, and the resulting complex can be recognized by HTL. Typically, antigenic peptides range in length from 12 to 20 amino acid residues, but peptides between 13 and 16 residues in length are frequently observed [54]. In fact, the 15-mers were the most abundant MHC II epitopes for *M. tuberculosis* and have been deposited in IEDB. As a result, we used the NetMHCIIpan-4.0 [51], [55] to predict the binding of 15-mer peptides to Human Leukocyte Antigen-DR (HLA-DR), HLA-DQ, HLA-DP and H-2–1 alleles. The prediction was also based on NNAlign_MA with % ranking < 2% and < 10% considered as strong and weak binders, respectively [51]. Also, we predicted 15-mer IFN-γ inducing epitopes for candidate proteins using the IFNepitope server [56], which uses a support vector machine hybrid approach that allows virtual screening of IFN-γ inducing peptide/epitope in a peptide library consisting of IFN-γ-inducible and non-inducible MHC II binders that activate T-helper cells. We then predicted the antigenicity of the IFN-γ inducing epitopes [45], and finally, we selected the 16 most promiscuous epitopes that were strong MHC-II binding, IFN-γ inducing, and antigenic.

It is important to note that signal peptides were removed from candidate proteins prior to the epitope prediction. In this study, signal peptides were screened using SignalP 5.0 [57] and TargetP-2.0 [58].

### 2.3 Linear B-cell epitope prediction

Linear B-cells epitopes (16-mers) were predicted using ABCpred [59], [60] with a default threshold of 0.51. Moreover, to increase the reliability of the prediction results, we also used BepiPred 2.0 [61] to predict linear B-cells epitopes. Epitopes obtained from these two softwares were further subjected to antigenicity prediction using VaxiJen2.0 [45]. Finally, we selected ten linear B-cell epitopes based on high ABCpred scores and antigenicity, with at least one epitope selected for each candidate protein.

### 2.4 Construction of the multi-epitope vaccine candidate with chimeric properties

The designed multi-epitope vaccine contains one HBHA (heparin-binding hemagglutinin) adjuvant, one Pan HLA DR-binding epitope (PADRE), 15 CTL, 16 HTL, 10 linear B-cells epitopes, and one His× 6 tag (Fig. 3). Linkers were used to join epitopes, prevent the production of junction epitopes, and enhance the procession and regeneration of individual epitopes in chimeric vaccines [62]. For the construction of this vaccine candidate, the HBHA adjuvant (UniProt ID: P9WIP9) was located at the N-terminus and linked to the downstream PADRE via an EAAAK linker. Then, the HTL epitopes joined by the GPGPG linkers were linked to PADRE. Moreover, CTL epitopes joined by AAY linker were connected to HTL epitopes via HEYGAEALERAG linker, which also joined CTL epitope unit to linear B-cell epitopes linked using KK linkers. Finally, a His× 6 tag was attached to the C-terminus of the chimeric protein.

### 2.5 Antigenicity, allergenicity and physicochemical properties

The antigenicity of the multiple-epitope vaccine and the eight component proteins were predicted by the VaxiJen 2.0 server [45], while the allergenicity of these proteins was predicted by the AllerTOP 2.0 server [63]. AllerTOP 2.0 uses amino acid E-descriptors, ACC transformation of protein sequences, and k-nearest neighbors (kNN) for allergen classification. The method achieved 85.3% accuracy with 5-fold cross-validation. For the prediction of physicochemical properties such as half-life, isoelectric point, instability index, aliphatic index, and grand average of hydropathicity (GRAVY) of this multiple-epitope vaccine, the ExPASy ProtParam server [64] was used. Further, the solubility of multi-epitope vaccine peptide was assessed using the proteinSol (PROSO II) server [65] based on a classifier exploiting the subtle differences between the well-known insoluble proteins from TargetDB and the soluble proteins from both TargetDB and PDB [66]. When evaluated using 10-fold cross-validation, it achieved 71.0% accuracy (area under ROC curve = 0.785).

### 2.6 Immune simulation

To characterize the immune response profile and immunogenicity of the vaccine, *in silico* immune simulations were performed using the C-ImmSim server [67]. C-ImmSim predicts immune interactions using position-specific scoring matrices derived from machine learning techniques for peptide prediction. It concurrently simulates three compartments representing three separate anatomical regions found in mammals: (i) the bone marrow, where hematopoietic stem cells were simulated to produce new lymphocytes and myeloid cells; (ii) the thymus, where naive T cells were selected to avoid autoimmunity; and (iii) the lymphatic organ such as lymph nodes. To effectively prime and boost the vaccine, we followed the approach of [68] where two injections were administered four weeks apart. All simulation parameters were set to default values, with time steps set to 10 and 94 (each time step is eight hours).

### 2.7 Disordered region prediction

Intrinsically disordered regions (IDRs) are present in many proteins. The disordered region was predicted using DISOPRED3 [69], which uses DISOPRED2 and two other machine-learning based modules trained on large IDRs to identify disordered residues. They were then annotated as protein-bound after using an additional SVM classifier [69].

### 2.8 Secondary and tertiary structure prediction

The secondary structure of the designed vaccine was predicted by the PSIPRED 4.0 server [70], which first uses PSI-BLAST to identify sequences closely related to the query protein. The tertiary structure of this vaccine was predicted using the Iterative Threading Assembly Refinement (I-TASSER) server [71]. There are four key steps in I-TASSER modeling; a) threading template identification; b) iterative structure assembly simulation; c) model selection and refinement; and d) structure-based functional annotation [72], [73]. I-TASSER generated five models, which were screened using ProSA-web [74], and the model with the lowest Z-score was selected for further refinement. ProSA-web compares the model scores obtained from experimentally verified structures deposited in PDB. A local quality score plot helps identify problematic areas in the model, and the same scores were represented using a color code on the presentation of the 3D structure. This is useful for early structural determination and refinement.

### 2.9 Tertiary structure refinement

The “coarse” 3D model of the vaccine candidate obtained by I-TASSER was refined in two steps using two servers; first with ModRefiner [75] followed by GalaxyRefine [76]. ModRefiner uses Cα traces to affect the construction and refinement of proteins obtained by two-step atomic-level energy minimization. First, the Cα traces were used to construct the main chain, followed by refinement of side chain rotamers and backbone atoms using physics- and knowledge-based composite force fields. GalaxyRefine utilizes multiple templates to generate reliable core structures, while unreliable loops or terminals were generated by optimization-based modeling.

### 2.10 Tertiary structure validation

The refined structure of the vaccine candidate was validated by Ramachandran plots generated from the PROCHECK [77] and MolProbity [78] databases. Ramachandran plots evaluate the backbone conformation of proteins by dividing amino acid residues into two regions: allowed and disallowed. PROCHECK utilizes stereochemistry to assess the net quality of protein structures by comparing them to the refined structures at the same resolution and then presenting regions requiring further analysis. Molprobity validates local and global macromolecule (proteins and nucleic acids) models by a mix of X-ray, NMR, computational, and cryoEM criteria [79]. The power and sensitivity to optimize hydrogen placement and all-atom contact analysis are widely used in an updated version of the covalent geometry and torsion angle criteria [80].

### 2.11 Discontinuous B-cell epitopes

Discontinuous B-cell epitopes in the native protein structure were predicted using ElliPro [81]. ElliPro implements three algorithms to approximate protein shape as an ellipsoid, calculates the residue protrusion index (PI), and clusters neighboring residues based on their PI values. ElliPro provides each output epitope with a score described as the averaged PI value for the epitope residue. An ellipsoid with a PI value of 0.9 consists of 90% of the contained protein residues, while the remaining 10% of residues lie outside the ellipsoid. For each epitope residue, the PI value is calculated from the centre of mass of residue lying outside the largest possible ellipsoid.

### 2.12 Molecular docking of chimeric proteins

Molecular docking of the designed vaccine (ligand) with Toll-Like Receptor-4 (TLR4) (PDB ID: 3FXI) immune receptor was performed using Patchdock [82]. The top 10 models were then refined using FireDock [83]. PatchDock replaces the Connolly dot surface representation of the molecules with concave, convex and flat patches. The models were then scored based on geometric fit and atomic desolvation. [82]. FireDock optimizes side chain conformations and orientation of the rigid-body and generates an output of a 3D refined complex based on the binding energy [83]. We selected the first model of Firedock based on global energy as the docking complex. Finally, the binding energy and dissociation content within the docking complex were predicted using the PRODIGY server [84].

### 2.13 Molecular dynamics simulation

Molecular dynamic simulations were performed on proteins using the fast and freely-accessible web-server, internal coordinates normal mode analysis server (iMODS) [85], and consistent and optimal docking result were obtained from the PatchDock-FireDock server. In internal coordinates, Normal Mode Analysis (NMA) generates collective motions critical for macromolecular function. iMODS presents mechanisms for exploring these modes as vibration analysis, motion animations, and morphing trajectories that were carried out almost interactively at different resolutions [85].

### 2.14 Reverse translation, codon optimization, and in silico cloning of the vaccine

To effectively express the vaccine candidate in *Escherichia coli* cells, cDNA was generated *in silico* through codon optimization and reverse translation using the Java Codon Adaptation Tool (JCAT) [86]. Optimization involved (i) avoiding rho-independent transcriptional terminators, ii) avoiding prokaryotic ribosome binding sites, (iii) avoiding cleavage site of restriction enzymes *Nco*I and *Xho*I, which serves as N-terminal and C-terminal restriction sites for the insertion of cDNA template of vaccine, and (iv) only partial optimization to apply site directed mutagenesis. Codon Adaptation Index (CAI) and GC content predicted the quality of the cDNA with an opal stop codon (TGA) inserted after the His× 6 tag. Then, the optimized DNA fragment of the chimeric vaccine candidate was integrated into the reverse strand of pET-28a(+) using the SnapGene tool [87].

### 2.15 Overview of the multi-epitope vaccine design

**Fig. 1:**
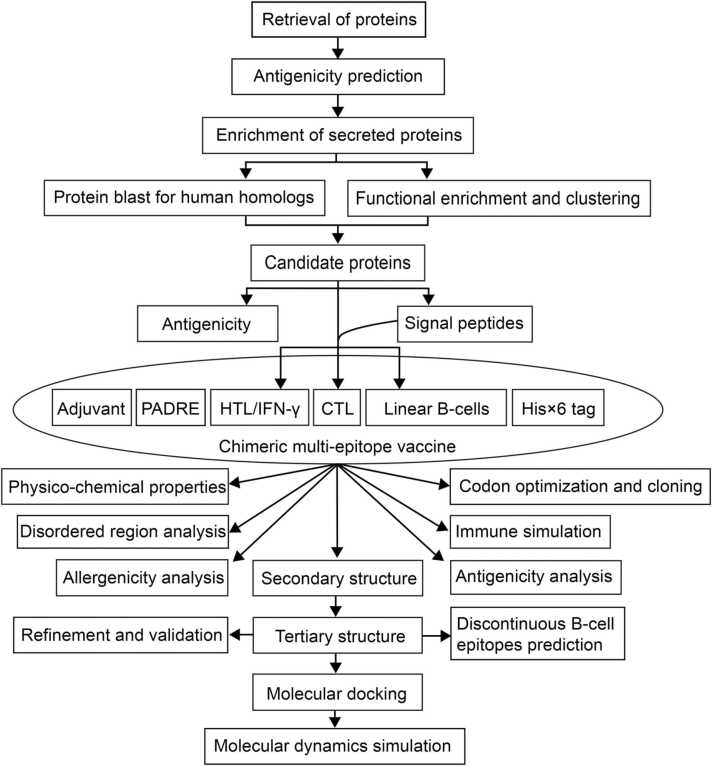
Overview of the multi-epitope vaccine design.

Fig. 1 summarized the overall concept of this multi-epitope vaccine design. Briefly, we analyzed the antigenicity of *M. tuberculosis* proteins obtained from IEDB and selected eight antigenic candidates for multi-epitope vaccine construction, considering their functional classification and subcellular localization. Then, we predicted the antigenic epitopes of these eight proteins and selected 15 CTL epitopes, 16 HTL epitopes, and 10 B-cells epitopes, which were further linked to HBHA adjuvant and PADRE through suitable linkers. Furthermore, we predicted the antigenicity, allergenicity, physicochemical properties, and immunogenic profile of the designed multi-epitope vaccine. Meanwhile, we also predicted the secondary and tertiary structures of the vaccine and analyzed its potential interaction with TLR4 immune receptor.

## 3 Results

### 3.1 Retrieval of *M. tuberculosis* proteins for multi-epitope vaccine construction

To construct a multi-epitope vaccine against TB, we first analyzed the antigenicity of proteins in *M. tuberculosis* H37Rv. A total of 492 proteins with validated immunological properties were obtained from the IEDB database, of which 402 proteins (81.70%) were predicted to be antigenic. For the functional characterization of these 492 proteins, 353 were enriched in the DAVID database, while 139 were not. Localization analysis showed that 97 of the 353 enriched proteins were localized in the extracellular compartment, the space outside the cell surface. Proteins localized in the extracellular compartment include outer membrane proteins and secreted proteins. Among the 97 proteins, 49 are secretion proteins. Further enrichment of 49 secreted proteins revealed that five proteins were secreted to the extracellular space and five were required for pathogenesis (experimentally validated by BioCyc) [48]. Moreover, when these ten proteins were aligned with the total proteins of *H. sapiens*, two proteins (Mpt83 and Mpt70) were highly similar to *H. sapiens* proteins. Therefore, the remaining eight proteins not similar to *H. sapiens* proteins (Table 1) were considered as final candidates for the construction of multi-epitope vaccine.

**Table 1:** General information about the eight selected proteins for multi-epitope vaccine construction.

Protein	UniProt ID	Function	Amino acid residue number	Allergenicity	Functional-annotation-based classification	References
EspA	P9WJE1	ESAT-6-system-1 (ESX-1) secretion-associated protein	404	Non-allergen	Secreted/pathogenesis	[88], [89]
Mpt63	P9WIP1	immunogenic protein	163	Non-allergen	Secreted/extracellular space	[90]
LprA	P9WK55	lipoprotein LprA	252	Allergen	Secreted/extracellular space	[91]
PPE18	L7N675	proline-proline-glutamic (PPE) family protein	403	Non-allergen	Secreted/pathogenesis	[92]
EsxA	P9WNK7	6 kDa early secretory antigen	97	Non-allergen	Secreted/pathogenesis	[93], [94]
EsxB	P9WNK5	10 kDa culture filtrate antigen	102	Non-allergen	Secreted/pathogenesis	[93]
LppX	P9WK65	phthiocerol dimycocerosate transporter	239	Non-allergen	Secreted/pathogenesis	[95]
EspC	P9WJD7	ESAT-6-system-1 (ESX-1) secretion-associated protein	105	Allergen	Secreted/pathogenesis	[89]
HBHA*	P9WIP9	heparin binding hemagglutinin	205	Non-allergen	Cell surface	[96]

* Indicates that the protein is used as adjuvant.

### 3.2 Prediction of T cell and B-cell epitopes of candidate proteins

**Fig. 2:**
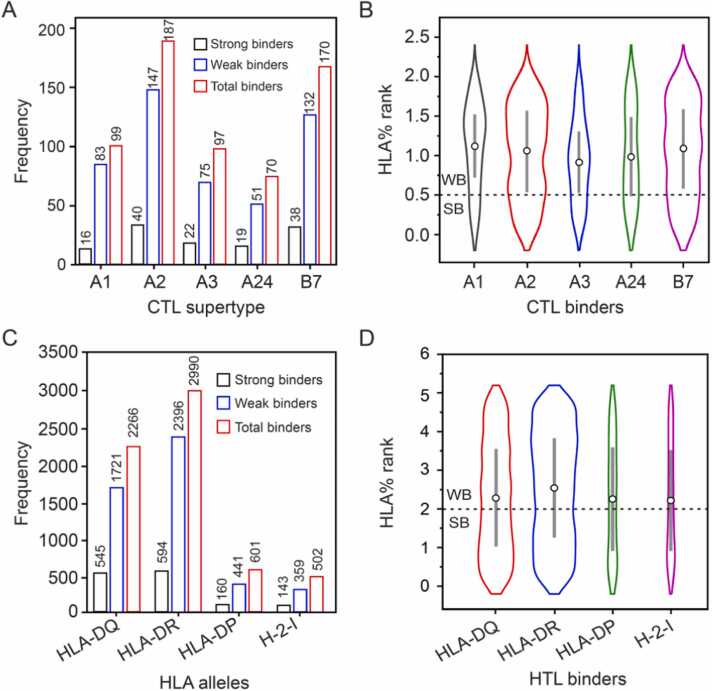
Distribution of CTL and HTL alleles among the candidate proteins. (**A**) Number and strength of binding epitopes of CTL alleles predicted by NetMHCpan-4.1. (**B**) Violin plot showing the distribution of CTL allele-binding epitopes based on percentage rank [A1 (HLA-A*01:01), A2 (HLA-A*02:01), A3(HLA-A*03:01), A24 (HLA-A*24:02), and B7 (HLA-B*07:02)]. (**C**) Number and strength of HTL allele-binding epitopes predicted by NetMHCIIpan-4.0. (**D**) Violin plot showing the distribution of binding epitopes based on percentage rank for human (HLA-DQ, HLA-DR, HLA-DP) and mouse (H-2-I) HTL alleles. SB and WB represent strong and weak binders, respectively.

Prior to epitope prediction, signal peptides were identified and deleted from candidate proteins. A total of 623 CTL epitopes from eight candidate proteins with 135 strong binders (16 of A1, 40 of A2, 22 of A3, 19 of A24, and 38 of B7 supertypes of MHC class I) were predicted by the NetMHCpan-4.1 server (Fig. 2A, B). Among the 135 strong binders, 36 were both antigenic and immunogenic. Finally, we selected 15 out of 36 CTL epitopes according to the following criteria: three epitopes per supertype and at least one per candidate protein (Note: EsxA had no strong binder that was both antigenic and immunogenic) (Table 2). Moreover, we predicted their HTL epitopes (MHC class II epitopes). 15-mer epitopes from four alleles (HLA_DRB, HLA_DP, HLA_DQ, and H-2-I) with a percentage ranking ≤ 2.0 were considered strong binders and ranked from the lowest to the highest score. The HLA-DR alleles had the highest number of MHC II binders (2990), while the HLA-DP had the least (601) (Fig. 2C, D). It should be noted that the immune response in granulomatous disorders such as tuberculosis, leprosis and sarcoidosis is dominated by IFN-γ producing T-helper-1 (Th1) cells [97]. In addition, previous reports revealed that the expression of Th1 cytokines (IFN-γ and IL-2) decreased in tuberculosis patients [98]. Therefore, to stimulate Th1 cells, we predicted 15-mer IFN-γ inducing peptides of these proteins. A total of 534 epitopes with IFN-γ inducing activity were predicted using NetMHCIIpan 4.0, of which 315 epitopes are antigenic. Of these 315 epitopes, 103 showed strong binding activity to at least one HLA allele. Also, polymorphism in HLA alleles results in allelic variation, leading to widely distinct peptide-binding specificities [55]. As a result, we selected one epitope with the strongest binding activity (most promiscuous epitope) from each of the eight candidate proteins. In addition, the remaining 95 epitopes were ranked based on binding strength, and then an additional eight epitopes were selected, with a maximum of two epitopes per candidate protein. Finally, we selected 16 out of 103 HTL epitopes according to the following criteria: at least one epitope and maximum three epitopes per candidate protein, strongest binding activity per candidate protein, and most antigenic and IFN-γ inducing. We then used them to construct this multi-epitope vaccine (Table 2).

**Table 2:** Selected epitopes for multi-epitope vaccine design.

Protein	CTL epitopes (% rank, IC 50, antigenicity, immunogenicity)	HTL epitopes (IFN-γ inducing activity, antigenicity)	Linear B-cell epitopes (ABCpred score, antigenicity)
EspA	FIIDPTISA (0.089, 17092.26, 1.261, 0.107)	DLTYIPVVGHALSAA (0.009, 0.554)	YSEGAAAGTEDAERAP (0.970, 1.195)
EspA	TYIPVVGHAL (0.101, 390.63, 0.500, 0.195)	ADGPVGAAAEQVGGQ (0.110, 1.395)	EDAERAPVEADAGGGQ (0.920, 1.640)
EspA	/	TTTKKYSEGAAAGTE (0.010, 1.508)	/
Mpt63	IPGYPVAGQVW (0.337, 416.95, 0.590, 0.033)	STGKIYFDVTGPSPT (2.000, 0.823)	GQVWEATATVNAIRGS (0.910, 0.466)
Mpt63	GPSPTIVAM (0.030, 48.29, 1.149, 0.156)	/	/
LprA	KSEDAKFVY (0.070, 625.66, 1.288, 0.013),	DAKFVYVDGHLYSDL (1.000, 0.403)	GGCSTEGDAGKASDTA (0.930, 2.554)
LprA	NKSEDAKFVY (0.127, 980.34, 1.114, 0.027)	/	LVQIQIAPTKDTSVTL (0.930, 0.851)
PPE18	VWGLTVGSW (0.353, 747.23, 0.689, 0.008)	AAQVRVAAAAYETAY (0.335, 0.642)	GARAGGGLSGVLRVPP (0.840, 1.358)
PPE18	APAAAAQAV (0.087, 11.84, 0.638, 0.052)	AMFGYAAATATATAT (0.604, 0.748)	/
PPE18	TPAARALPL (0.015, 6.38,0.434, 0.123)	QNGVRAMSSLGSSLG (0.458, 0.523)	/
EsxA	/	EQQWNFAGIEAAASA (2.000, 0.742)	GGSGSEAYQGVQQKWD (0.860, 1.801)
EsxA	/	AGIEAAASAIQGNVT (2.000, 0.937)	/
EsxB	STNIRQAGVQY (0.224, 1137.27, 1.111, 0.107)	AVVRFQEAANKQKQE (0.230, 0.858)	LKTQIDQVESTAGSLQ (0.860, 1.039)
EsxB	/	STNIRQAGVQYSRAD (1.000, 0.999)	/
LppX	VLDPAAGVTQL (0.107, 4393.17, 0.426, 0.1090	QGVPFRVQGDNISVK (0.385, 1.992)	SLLGITSADVDVRANP (0.950, 1.206)
LppX	HVAVRTTGK (0.217, 38.73, 0.994, 0.194)	STTKITGTIPASSVK (1.000, 0.529)	/
LppX	RVQGDNISVK (0.155, 237.77, 2.643, 0.021)	/	/
EspC	SQFNDTLNV (0.185, 38.23, 0.616, 0.076)	TAGVDLAKSLRIAAK (0.030, 0.670)	AVDASSGVEAAAGLGE (0.890, 1.188
EspC	SLHTAGVDLAK (0.385, 559.6, 1.024, 0.195)	RIAAKIYSEADEAWR (0.027, 0.456)	/

As for linear B-cell epitope prediction, two servers (BepiPred2.0 and ABCpred) were used and 166 epitopes were obtained. We predicted the antigenicity of the top five epitopes for each protein, and then selected ten antigenic epitopes (16-mer, at least one from each candidate protein) (Table 2).

### 3.3 Multi-epitope vaccine construction

**Fig. 3:**
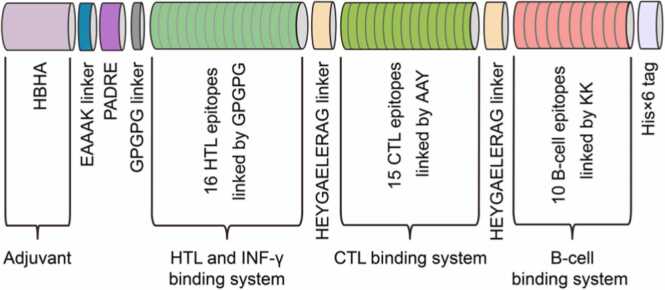
Schematic representation of multi-epitope vaccine protein.

For multi-epitope vaccine construction, HBHA, PADRE, 16 HTL (15-mer), 15 CTL and 10 linear B-cell (16-mer) epitopes were connected via appropriate linkers and tailed by a His× 6 tag (Fig. 3). Adjuvants are important components of vaccines that can boost innate and adaptive immune responses [99]. Many proteins from *M. tuberculosis* have adjuvant properties and have been used to design vaccine [17], [27], [87], [100], [101], [102], [103], [104], [105]. One of the most widely used is HBHA [100], [101], [102], [103], [104], [105], which is a potent immunostimulant that induces dendritic cells (DCs) maturation through TLR4 processing [105], [106]. DCs treated with HBHA have been found to activate naive T cells, polarize CD4^+^ and CD8^+^ T cells to secrete IFN-γ, and induce T-cell-mediated cytotoxicity [47]. In this study, we placed HBHA as a protein adjuvant at the N-terminus because it is suitable to initiate the synthesis of the vaccine construct from HBHA, a naturally occurring protein. HBHA has previously been placed at the N-terminus of some multi-epitope vaccines as adjuvant [105], [107]. In addition to adjuvants, PADRE, which is safe for humans, can significantly enhance vaccine immunogenicity because it binds with high affinity to multiple mouse and human MHC-II alleles with high affinity to induce Th cell-mediated responses [47], [62]. HBHA and PADRE were connected by a helical EAAAK linker, which provides rigidity while separating the two protein components to enhance efficiency and reduce interference. Further, a Glu-Lys salt bridge formed within the segments stabilized the linker.

Since PADRE can enhance the activity of vaccine HTL epitopes [54], we placed the 16 HTL epitopes after PADRE. The GPGPG linker was used to link HTL epitopes to each other and to PADRE since it facilitates immune cell progression and epitope presentation [108], [109]. Moreover, GPGPG induces HTL immune response, which can disrupt junctional immunogenicity and restore the immunogenicity of individual epitopes after processing [108]. For the 15 CTL epitopes, the individual epitope was joined by AAY linker, which helps the epitope produce suitable sites to bind to the TAP transporter and enhance epitope presentation [47]. Further, the entire CTL epitope subunit was linked to the upstream HTL epitopes via the HEYGAEALERAG linker that possesses five appropriate cleavage sites (A7-L8, A5-E6, Y3-G4, R10-A11, and L8-E9) essential for eukaryotic proteasomal and lysosomal degradation systems. In eukaryotes, proteasome and lysosomes are the most important proteolytic machineries, which utilize the ubiquitin-proteasome system (UPS) and autophagy pathway, respectively [110]. HEYGAEALERAG linker was also used to link the CTL epitope subunit to the 10 downstream linear B-cell epitopes linked together by KK linkers. According to previous studies, the KK linker prevents the induction of antibodies into the amino acid sequence resulting from the combination of the two peptides, thereby facilitating the specific display of each peptide to antibody [111]. Finally, a His× 6 tag was attached to the C-terminus of the last linear B-cell epitope for subsequent purification and characterization of the vaccine (Fig. 3).

The final chimeric protein consists of 933 amino acid residues, starting with HBHA adjuvant, followed by 41 epitopes, and ending with a His× 6 tag (Fig. 3). Since this chimeric protein is composed of multi-epitope antigens from eight proteins, we named it MTBV8, the Multi-epitope TB Vaccine derived from **8** candidate proteins.

### 3.4 Analysis of the antigenicity, allergenicity, and physiochemical parameters of MTBV8

**Fig. 4:**
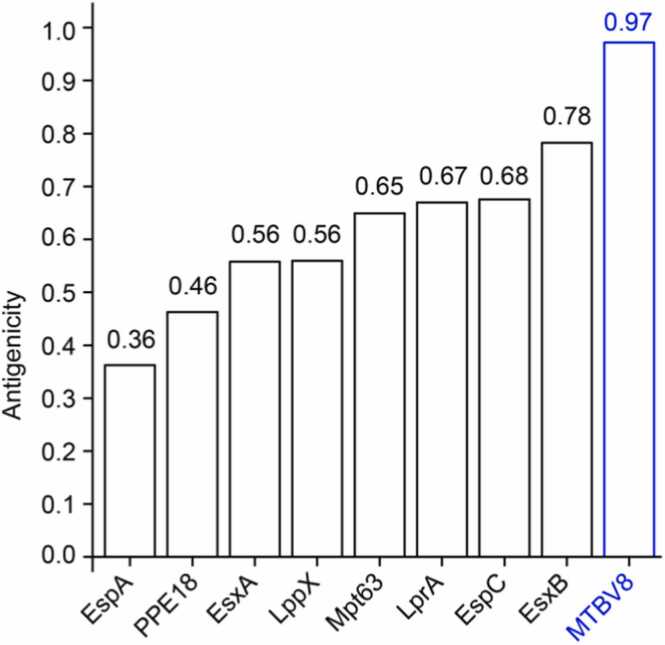
Antigenicity of the designed vaccine MTBV8 and other candidate proteins.

Previous studies have demonstrated that antigenicity is required for human vaccines in order to elicit humoral immune response, leading to the generation of memory cells directed against epitopes of infectious agent [112]. Therefore, we predicted the antigenicity of the multi-epitope vaccine MTBV8 using the VaxiJen 2.0 server and obtained an antigenicity of 0.97, which is significantly higher than the threshold value (0.4) [45], [46] for a bacterial protein to be considered antigenic. Notably, MTBV8 was more antigenic than all component proteins (Fig. 4), suggesting that it could effectively stimulate host immune responses.

Moreover, many microbial macromolecules have been reported to have the potential to induce hypersensitivity reactions in humans [113]. To analyze hypersensitivity issue of MTBV8 and the eight constituent proteins, we predicted their allergenicity using the AllerTOP 2.0 server. Two of the eight component proteins (LprA and EspC) were predicted to be allergens (Table 1). It should be noted that although the designed multi-epitope vaccine (MTBV8) contained ten epitopes obtained from these two proteins, it was predicted to be non-allergic, indicating that this vaccine is safe.

To further characterize MTBV8, we analyzed its physicochemical properties through the ExPASy ProtParam server. The recombinant protein has a molecular weight of 94.84 kDa and an isoelectric point of 8.91. Moreover, the protein was considered stable with a calculated instability index (II) of 26.44 (proteins with instability index > 40 were considered unstable) [64]. Estimated *in vitro* half-life is 30 h in mammalian reticulocytes, longer than *in vivo* 20 h in yeast, and *in vivo* 10 h in *E. coli*. In addition, the aliphatic index of MTBV8 was 71.91, and the overall average value of hydrophilicity (GRAVY) was − 0.32, indicating that the recombinant protein was hydrophilic. Finally, the proportional solubility value for MTBV8 (0.54) was greater than the average for the population dataset (0.45), indicating that the vaccine candidate was more soluble than half of the *E. coli* proteins [114]. The above results indicated that MTBV8 has good physical and chemical properties and is suitable for use as a vaccine.

### 3.5 Immunogenic profile of MTBV8

**Fig. 5:**
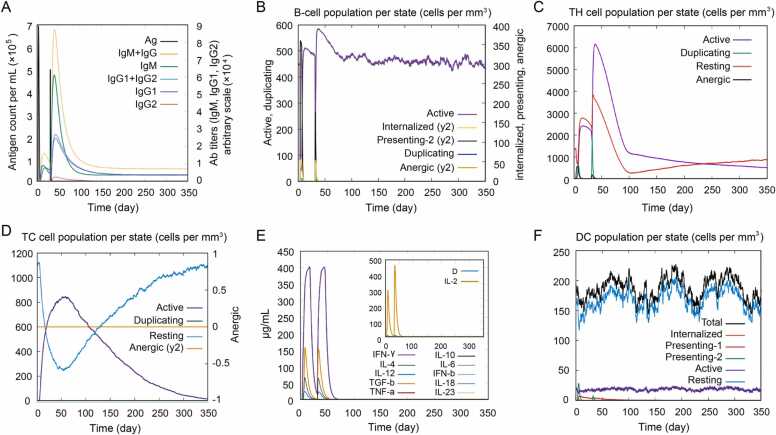
Immunogenicity profile of MTBV8. Two injections were administered on day 1, and day 29, respectively (4 weeks apart). (**A**) Production and changes of different immunoglobulins in response to MTBV8 injection; (**B**) B lymphocyte counts for each entity state after two injections; (**C**) CD4^+^ T-helper (TH) lymphocyte counts for each entity state after two injections; (**D**) CD8^+^ T-cytotoxic (Tc) lymphocyte counts for each entity state after two injections; (**E**) Changes in cytokine and interleukin concentrations after two injections; “D” in the built-in diagram represents the insertion point of the danger signal; (**F**) DC counts for each entity state after two injections.

To assess the immunogenicity profile of MTBV8, we analyzed the immune responses *in silico* via the C-IMMSIM immune server. As shown in Fig. 5A, the IgM and IgG1 titers were substantially increased after secondary immune stimulation by MTBV8. Moreover, the level of active B-cells increased and remained at high level after each immunization (Fig. 5B). Even for B cells in plasma, the B isotypes IgM and IgG1 remained high after immunization (Supplementary Fig. 1A, B). As for the effect on T cells, the levels of Th memory cells (y2) (Supplementary Fig. 1C) and active Th cells (Fig. 5C) increased rapidly after primary immunization and were significantly boosted after secondary immunization. Regulatory T cells (primarily active cells) were stimulated upon initial immunization and then declined rapidly (Supplementary Fig. 1D). Interestingly, the level of active cytotoxic T (Tc) cells increased rapidly after primary immunization, remained high in the second immunization, and then declined steadily, whereas resting Tc cells showed the reverse trend to active Tc cells (Fig. 5D). In contrast, the levels of anergic (y2) Tc cells (Fig. 5D) and memory Tc cells (Supplementary Fig. 1E) remained unchanged upon immunization. In addition, it should be noted that the levels of the immune factors IFN-γ and IL-2 secreted by T cells that are critical for the immune response against *M. tuberculosis* [115] also significantly increased after MTBV8 immunization (Fig. 5E).

Furthermore, we predicted the effect of MTBV8 vaccination on innate immune cell populations (Fig. 5F and Supplementary Fig. 1F-H). Presenting-2 cells in DCs and macrophages increased rapidly upon the first immunization, whereas only a small increase was observed after the second immunization (Fig. 5F and Supplementary Fig. 1G). In addition, in macrophages, the first immunization resulted in a simultaneous increase or decrease in active and resting macrophages. A few weeks after the second immunization, active macrophages decreased rapidly while resting macrophages increased rapidly. (Supplementary Fig. 1G). Notably, natural killer cells and active epithelial cell populations exhibited a fairly constant pattern upon immunization. (Supplementary Fig. 1F, H). Taken together, these predictions suggested that MTBV8 can potently stimulate both innate and adaptive immune responses, making it a potentially effective vaccine candidate.

### 3.6 Predicted secondary and tertiary structures of MTBV8

To better characterize MTBV8, we predicted its secondary and tertiary structures, followed by refining its tertiary structure. The secondary structure of MTBV8 consisted of 42.12% helices, 11.79% strands and 46.09% coils (Supplementary Fig. 2). According to DISOPRED3 predictions, 27.11% of amino acid residues were disordered.

The tertiary structure of MTBV8 was predicted by the I-TASSER server, and 5 models with good *C*-scores were obtained. The *C*-score is between −5.0 and 2.0 and is proportional to the reliability of the prediction. These models were subjected to ProSA-web analysis and model 4 was selected due to the following characteristics; C score = −3.8, Z score = −4.4. Ramachandran plot analysis of this tertiary structure showed that less than 70.0% of the residues were in preferred regions, thus, further refinement of the tertiary structure is required.

**Fig. 6:**
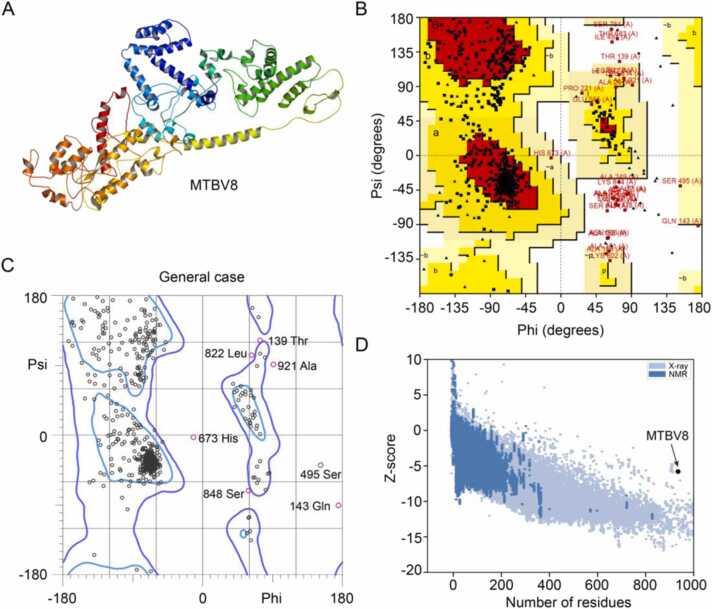
Tertiary structure of MTBV8 predication and its validation. (**A**) Refined tertiary structure of MTBV8; (**B**) Ramachandran plots of tertiary structure obtained from ProCheck; (**C**) Ramachandran plots of tertiary structure obtained from Molprobity; (**D**) Validation of the refined structure of MTBV8 with a Z-score of − 5.76 using ProSA-web.

The tertiary structure obtained by I-TASSER was refined by the ModRefiner server, followed by the GalaxyRefine server to generate five models. Among the five refined models, model 2 had the best structure when considering the following parameters: Global distance test high accuracy (0.89), RMSD (0.58), MolProbity (2.31), clash score (14.8), poor rotamer score (0.80) and Ramachandran plot score (86.5%) (Fig. 6A). Ramachandran plot analysis of this structure using PROCHECK revealed that of the 753 non-glycine, non-proline, and non-terminal residues, 78.9% of them were in the most favorable region [A, B, L], 17.3% were in the additionally allowed region [a, b, l, p], and 1.5% in the generally allowed region [∼a, ∼b, ∼l, ∼p], with only 2.4% in the disallowed region (Fig. 6B). When analyzing the Ramachandran plot of the refined structure using Molprobity, we found that 86.5% of the amino acid residues were located in the favorable region, which was the same as obtained by the GalaxyRefine server (Fig. 6C). Furthermore, 98.0% of the residues were in the allowable region, while 19 residues were outliers (phi, psi) (Fig. 6C)**.** Although the refined model has more residues in the favored region compared to the model generated by I-TASSER, it needs further validation.

Model validation is a key step in the model building process as it can identify potential errors in predicted 3D models [116]. To validate the refined model of MTBV8, ProSA-web and ERRAT analyses were performed. ProSA-web analysis showed a Z-score of − 5.76 for MTBV8 (Fig. 6**D**), which was slightly outside the experimentally determined range of scores for proteins of similar size. However, ERRAT analysis showed an overall quality factor of 70.94 for refined MTBV8. Since an ERRAT score greater than 50 represents good quality model [117], a score of 70.94 indicated that we have high confidence in the modeled structure for subsequent analysis.

For discontinuous conformational B-cell epitopes, we predicted 10 of them, with scores ranging from 0.85 to 0.53 (Supplementary Table 1).

### 3.7 Molecular docking of MTBV8 with immune cell receptors

**Fig. 7:**
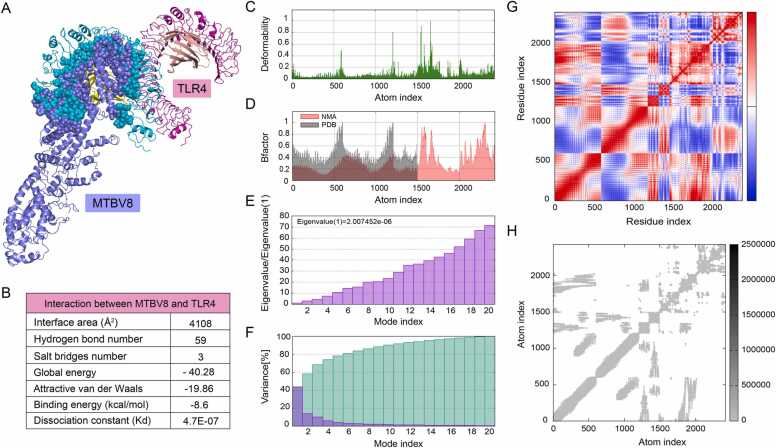
Molecular docking and dynamic simulation of MTBV8 with immune receptor TLR4. (**A**) Potential interaction of MTBV8 with TLR4. The overall structure of MTBV8 and TLR4 is shown in cartons, while the interface region is shown in spheres. (**B**) Detailed interaction parameters between MTBV8 and TLR4. (C) Deformability, (D) B factor (E) Eigen value, (F) Variance, (G) Covariance map: correlated (red), uncorrelated (white), anti-correlated (blue), (H) Elastic network (dark grey indicate more rigid regions).

The strong affinity of vaccines for immune cell receptors produces a stable immune response [118]. To gain insight into the potential interactions between MTBV8 and immune cell receptors, we carried out molecular docking between MTBV8 and the immune receptor TLR4 (Fig. 7A and B). Previous studies have shown that TLR4 ligands can activate DCs, which in turn activate naive T cells, effectively polarize T cells (CD4^+^ and CD8^+^ cells) to secrete IFN-γ, and induce T cell-mediated-cytotoxicity [106]. These processes would lead to an increase in the pool of effector memory cells [119]. Since the interaction of TLR4 with antigenic peptides leads to responses against TB infection, we focused on the interactions between MTBV8 and TLR4 (Fig. 7A). Molecular docking results from Firedock showed that the most favored refined model had a global energy of − 40.28, an attractive van der Waals energy (aVdW) of − 19.86, a repulsive energy (rVdW) of 2.19, an atomic contact energy of 4.70 and a hydrogen bond (HB) contribution of − 1.03 (Fig. 7B). Additionally, Prodigy predicted a binding energy between TLR4 and MTBV8 of − 8.6 kcal/mol. The results above indicated that MTBV8 has a strong affinity to TLR4, enabling it to generate a stable immune response.

### 3.8 Molecular dynamics simulation

The molecular dynamic simulation was used to analyze the motion of atoms in the vaccine [120]. Main chain deformability reveals the extent to which a molecule can deform at each constituent residue. Regions of high deformation indicated the position of the chain “hinge” (Fig. 7C), and experimental B factor was obtained from the corresponding PDB data bank while the calculation was performed by multiplying the NMA mobility by 8pi^2 (Fig. 7D). The eigenvalue assigned to each normal mode is a measure of motion stiffness, which is directly related to deformation energy of the entire structure. The eigenvalue is directly proportional to the ease of deformation (Fig. 7E). The variance of each normal mode is inversely proportional to the eigenvalue. Individual variances are colored in brown while cumulative variances are colored in green) (Fig. 7F). In the covariance matrix, there is coupling between pairs of residues, and correlated (red), uncorrelated (white) and anti-correlated (blue) motions were displayed (Fig. 7G). Elasticity network represented pairs of atoms connected by springs, and each dot represented a spring between corresponding pairs of atoms. The color of the dots signified their stiffness; darker gray dots illustrate stiffer springs and vice versa (Fig. 7H). Molecular dynamics simulations using the iMODS server suggested that the docking complex between MTBV8 and TLR4 was stable.

### 3.9 In silico optimization and cloning of MTBV8

Protein synthesis is a prerequisite for its activity [121]. In line with this, increased transcriptional and translational efficiencies of the vaccine are necessary for its overexpression in *E. coli* cells via a self-replicating plasmid pET28a(+) [103]. Its optimization was thereof achieved by the Codon Adaptation Index (CAI) of 0.99, and a GC content (54.81%) between 30% and 70%, which are generally considered favorable for protein expression in host. The DNA fragment of *mtbv8* could be cloned into pET-28a(+) vector between the *Nco*I and *Xho*I restriction sites using SnapGene to generate a pET-28a(+)-*mtbv8* recombinant plasmid (Supplementary Fig.3).

## 4 Discussion

### 4.1 The emergence of multi-epitope vaccines against tuberculosis

Vaccination is the most reliable way to fight tuberculosis infection. For many years, scientists have identified numerous promising vaccine candidates that could replace BCG, the only approved TB vaccine [8], [100], [122], [123], [124], [125]. Recently, recombinant TB vaccines have received increasing attention. Several recombinant vaccines have demonstrated efficacy in preclinical and clinical trials, including the ID93/GLA-SE consisting of four vaccine candidates Rv2608 (PE/PPE family), Rv1813 (expressed under stress/hypoxia), ESXV, and ESXW, which are promising in mice pre-clinical trial [122]. For clinical trials, GamTBvac, obtained by fusing Ag85a and ESAT6-CFP10, was successful in phase I trials [125], while M72, consisting of two candidates [Mtb39A (PPE18) and Mtb32A], yielded significant potency in phase II trials [123]. Successful trials of these recombinant vaccines indicates the greater potency of epitope-based vaccines that simply incorporates the immunological properties of different candidate proteins into a synthetic protein [124]. Notably, several TB subunit vaccines have been derived from a large number of vaccine candidates, and they have emerged as potentially attractive candidates in animal model studies [100]. For instance, MP3RT, a multi-epitope peptide TB vaccine candidate consisting of six immunogenic HTL peptides, induced significantly higher levels of IFN-γ and CD3^+^IFN-γ^+^ T lymphocytes and lower colony forming units (CFUs) in the lung and spleen of humanized mice than wild-type mice [8]. Going forward, the research for greater potency in these vaccines has led to an enrichment of potential protein candidates. Further, proteins secreted by *M. tuberculosis* have also been considered in the design of MTBV8 (Table 1), because they are important for TB pathogenesis and virulence [126]. In addition, some secreted proteins of the outer layers of *M. tuberculosis* cells can serve as useful antigens. For instance, EsxA and EsxB have been purified from the capsule of *M. tuberculosis* [127] and retention of EsxA in the capsule has been implicated in cytotoxicity [128]. In addition, EsxA is required for bacterial cell wall integrity [129]. Overall, secreted proteins have sufficiently exposed surfaces such that they are targets of host immune responses [130], [131].

### 4.2 Potential stimulation of the innate immune system by MTBV8

Screening for epitope in candidate proteins involves activation of the innate immune system followed by antigen recognition by lymphocytes, because both factors are required for the subsequent activation of the adaptive immune system [50]. For the innate response, the formation of a stable complex between MTBV8 (aided by the TLR4 stimulator HBHA as an adjuvant) and TLR4 of DCs (Fig. 5F) is important because TLR4 ligands can activate DCs and activated DCs can in turn activate naive T cells, effectively polarize CD4^+^ and CD8^+^ T cells to secrete IFN-γ, and induce T cell-mediated cytotoxicity [106] and subsequently increased pool of effector memory cells [119]. This complex likely contributed to the increase in memory cell (y2) population in B lymphocytes (Supplementary Fig. 1A) and Th lymphocytes (Supplementary Fig. 1C) compared to non-memory cells. Immune stimulation by MTBV8 is comparable to that obtained with other multiepitope vaccines against TB [33], [37], [39] and other diseases [27], [62], [107], [124], [132]. This hinted at the consistency of the multi-epitope vaccine mechanism.

### 4.3 Potential stimulation of adaptive immune system by MTBV8

B and T lymphocytes are the primary effector cells that coordinate adaptive immune responses through humoral or cell-mediated immunity by recognizing parts of invading pathogen, referred to as antigens [50]. To induce a humoral adaptive immune responses, we incorporated high scoring B-cell epitopes (Table 2) for recognition and destruction by antibodies expressed and secreted by B lymphocytes [50], [133]. Vaccine stimulation of active B lymphocytes (Fig. 5B), mostly IgM- and IgG1-secreting B cells (Supplementary Fig. 1A), may result in excessive secretion of IgM and IgG1 (Fig. 5A). The high antigenicity of the vaccine (Fig. 4) may have important implications for enhancing antibody responses (Fig. 5A). Indeed, cell-mediated immune responses are highly dependent on the ability of T cells to recognize antigens [134]. To achieve this, we selected strong antigenic and strong MHC-binding CTL and HTL epitopes (Table 2), which enhanced peptide:MHC complex (pMHC) stability, which is the key factor controlling MHC peptide immunogenicity [135] and plays a role in the priming of CD8^+^ and CD4^+^ T cells by DCs, leading to T lymphocyte responses (Fig. 5C, Supplementary Fig. 1C) [136]. The incorporation of appropriate linkers and synthetic oligopeptides (PADRE and HEYGAEALERAG) and precise positioning of each component could facilitate epitope procession and presentation (Fig. 3). Due to allele variation among humans, we selected CTL epitopes based on recognition of MHC-I alleles of major human races Supplementary Table 1(Table 2), and promiscuous HTL epitopes were selected after screening for IFN-γ inducing properties and antigenicity.

### 4.4 The development prospects of MTBV8

This *in silico* vaccine design is a relevant indicator in the screening phases in terms of the synthesis and efficacy of candidate vaccines. For synthesis, our predictions revealed that it can be synthesized in the *E. coli* system, however, other systems such as insect and mammalian cells could also be considered if *E. coli*-based systems present difficulties. Wet laboratory validation is the next stage in confirming vaccine candidates after *in silico* studies. This method has actually been used effectively in some vaccine candidates, which were also designed first *in silico* and later proved effective in the experimental phase [137].

## 5 Conclusion

A key support of the Sustainable Development Goal of WHO for the complete eradication of TB is access to an potent vaccine to prevent *M. tuberculosis* infection. Previous studies have shown that chimeric multi-epitope vaccines can exploit the immune/antigenic properties of certain proteins to enhance immune efficacy. In this study, we used an *in silico* approach to design a chimeric vaccine (MTBV8) by integrating 41 promiscuous epitopes derived from eight antigenic proteins secreted by *M. tuberculosis* H37Rv. MTBV8 was predicted to be stable, soluble, safe, highly antigenic and immunogenic. Immuno-informatics analysis showed that MTBV8 exhibited good affinity for the major immune cell receptor TLR4. Importantly, vaccination stimulation of MTBV8 could significantly increase the levels of B cells, T cells, and innate immune cell populations and stimulated the production of immunoglobulins (IgG1, IgM, etc.) as well as immune factors (IFN-γ, IL-2, etc). Taken together, the multi-epitope vaccine MTBV8 is a relevant indicator in the design phase of anti-TB vaccines and may become a potential cornerstone in the realization of the ‘The End TB strategy’.

## CRediT authorship contribution statement

**Binda T. Andongma:** Conceptualization, Methodology, Software, Writing – original draft. **Yazheng Huang:** Data curation, Software. **Fang Chen:** Software, Visualization. **Qing Tang:** Data curation, Formal analysis. **Min Yang:** Software, Visualization. **Shan-Ho Chou:** Writing – review & editing. **Xinfeng Li:** Conceptualization, Methodology, Software, Funding acquisition. **Jin He:** Supervision, Writing – review & editing, Funding acquisition.

## Declaration of Competing Interest

The authors declare that they have no known competing financial interests or personal relationships that could have appeared to influence the work reported in this paper.
