# Supplementary material for: In silico design of a promiscuous chimeric multi-epitope vaccine against Mycobacterium tuberculosis
Source: Comput Struct Biotechnol J. 2023 Jan 16;21:991–1004. doi: 10.1016/j.csbj.2023.01.019 (PMC9883148; doi:10.1016/j.csbj.2023.01.019)
Supplement: Supplementary file 1 — Supplementary material [file mmc1.docx]

***In silico* design of a promiscuous chimeric multi-epitope vaccine against *Mycobacterium tuberculosis***

**Supplementary Table 1. Discontinuous B-cell epitopes of MTBV8**

| **No.** | **Residues** | **Number of residues** | **Score** |
| --- | --- | --- | --- |
| 1 | :R38, _:A39, _:E40, _:E41, _:T42, _:R43, _:T44, _:D45, _:T46, _:R47, _:S48, _:R49, _:V50, _:E51, _:E52, _:S53, _:R54, _:A55, _:R56, _:L57, _:T58, _:K59, _:L60, _:Q61, _:E62, _:D63, _:L64, _:P65, _:E66, _:Q67, _:L68, _:T69, _:E70, _:L71, _:R72, _:E73, _:K74, _:F75, _:T76, _:A77, _:E78, _:E79, _:L80, _:R81, _:K82, _:A83, _:A84, _:E85, _:G86, _:Y87, _:L88, _:E89, _:A90, _:A91, _:T92, _:S93, _:R94, _:Y95, _:N96, _:E97, _:L98, _:V99, _:E100, _:R101, _:G102, _:E103, _:A104, _:A105, _:L106, _:E107, _:R108, _:L109, _:S111, _:Q112, _:Q113, _:S114, _:F115, _:E116, _:E117, _:V118 | 80 | 0.849 |
| 2 | _:G318, _:P319, _:G320, _:P321, _:G322, _:A323, _:A324, _:V326, _:R327, _:G414, _:N415, _:V416, _:T417, _:G418, _:P419, _:G420, _:P421, _:G422, _:A423, _:V424, _:V425, _:R426, _:F427, _:Q428, _:E429, _:A430, _:A431, _:N432, _:K433, _:Q434, _:K435, _:Q436, _:E437, _:G438, _:P439, _:G440, _:P441, _:G442, _:S443, _:T444, _:N445, _:I446, _:Q448, _:A449, _:G450, _:V451, _:Q452, _:Y453, _:S454, _:R455, _:A456, _:D457, _:G458, _:P459, _:G460, _:P461, _:G462, _:Q463, _:G464, _:V465, _:P466, _:F467, _:R468, _:V469, _:Q470, _:G471, _:D472, _:N473, _:I474, _:S475, _:V476, _:K477, _:G478, _:P479, _:G480, _:P481, _:G482, _:S483, _:T484, _:T485, _:K486, _:I487, _:T488, _:G489, _:T490, _:I491, _:P492, _:A493, _:S494, _:S495, _:V496, _:K497, _:G498, _:P499, _:G500, _:P501, _:G502, _:T503, _:A504, _:G505, _:V506, _:D507, _:L508, _:A509, _:K510, _:S511, _:L512, _:R513, _:I514, _:A515, _:A516, _:K517, _:G518, _:P519, _:G520, _:P521, _:G522, _:R523, _:I524, _:A525, _:A526, _:K527, _:I528, _:E531 | 124 | 0.812 |
| 3 | :A164, _:P165, _:A166, _:K167, _:K168, _:A169, _:A170, _:P171, _:A172, _:K173, _:K174, _:A175, _:A176, _:P177, _:A178, _:K179, _:K180, _:A181, _:A182, _:A183, _:K184, _:K185, _:A186, _:P187, _:A188, _:K189, _:K190, _:A191, _:A192, _:A193, _:K194, _:K195, _:V196, _:T197, _:Q198 | 35 | 0.693 |
| 4 | _:N592, _:D593, _:R639, _:V640, _:Q641, _:G642, _:D643, _:N644, _:I645, _:S646, _:V647, _:K648, _:A649, _:A650, _:Y651, _:S652, _:L653, _:H654, _:T655, _:A656, _:A703, _:A705, _:A707, _:A708, _:A709, _:A710, _:Q711, _:A712, _:V713, _:A714, _:A715, _:Y716, _:T717, _:P718, _:A719, _:A720, _:R721, _:A722, _:L723, _:P724, _:L725, _:A726, _:A727, _:Y728, _:G729, _:P730, _:S731, _:P732, _:T733, _:I734, _:V735, _:A736, _:M737, _:H738, _:E739, _:Y740, _:G741, _:A742, _:E743, _:A744, _:L745, _:E746, _:R747, _:G749, _:G753, _:A754, _:A755, _:A756, _:G757, _:T758, _:E759, _:D760, _:A761, _:E762, _:R763, _:A764, _:P765, _:K766, _:K767, _:D769, _:A770, _:E771, _:A777, _:D778, _:A779, _:G780, _:G781, _:G782, _:Q783, _:K784, _:K785, _:G786, _:Q787, _:V788, _:W789, _:E790, _:A791, _:T792, _:A793, _:T794, _:V795, _:N796, _:A797, _:I798, _:R799, _:G800, _:S801, _:K802, _:K803, _:G804, _:G805, _:C806, _:S807, _:T808, _:E809, _:G810, _:D811, _:A812, _:G813, _:K814, _:A815, _:S816, _:D817, _:T818, _:A819, _:K820, _:K821, _:L822, _:V823, _:Q824, _:I825, _:Q826, _:I827, _:A828, _:P829, _:T830, _:K831, _:D832, _:S834, _:V835, _:T836, _:L837, _:K838, _:K839, _:G840, _:A841, _:R842, _:A843, _:G844, _:G845, _:G846, _:L847, _:S848, _:G849, _:V850, _:L851, _:R852, _:V853, _:P854, _:P855, _:K856, _:K857, _:G858, _:G859, _:S860, _:G861 | 183 | 0.676 |
| 5 | _:D290, _:V291, _:T292, _:G293, _:P294, _:S295, _:P296 | 7 | 0.617 |
| 6 | :A336, _:P339, _:G340, _:P341, _:G342, _:A343, _:M344, _:F345, _:G346, _:Y347, _:A348, _:A349, _:A350, _:T351, _:A352, _:T353, _:A354, _:T355, _:A356, _:T357, _:G358, _:P359, _:G360, _:P361, _:G362, _:Q363, _:N364, _:G365, _:A368, _:M369, _:L372, _:A407 | 32 | 0.579 |
| 7 | _:V913, _:D914, _:H929, _:H930, _:H931, _:H932, _:H933 | 7 | 0.56 |
| 8 | _:N908, _:P909, _:K910 | 3 | 0.548 |
| 9 | _:G311, _:H312, _:S315, _:D316 | 4 | 0.536 |
| 10 | _:G924, _:L925, _:G926, _:E927 | 4 | 0.53 |

**Supplementary figures**

**
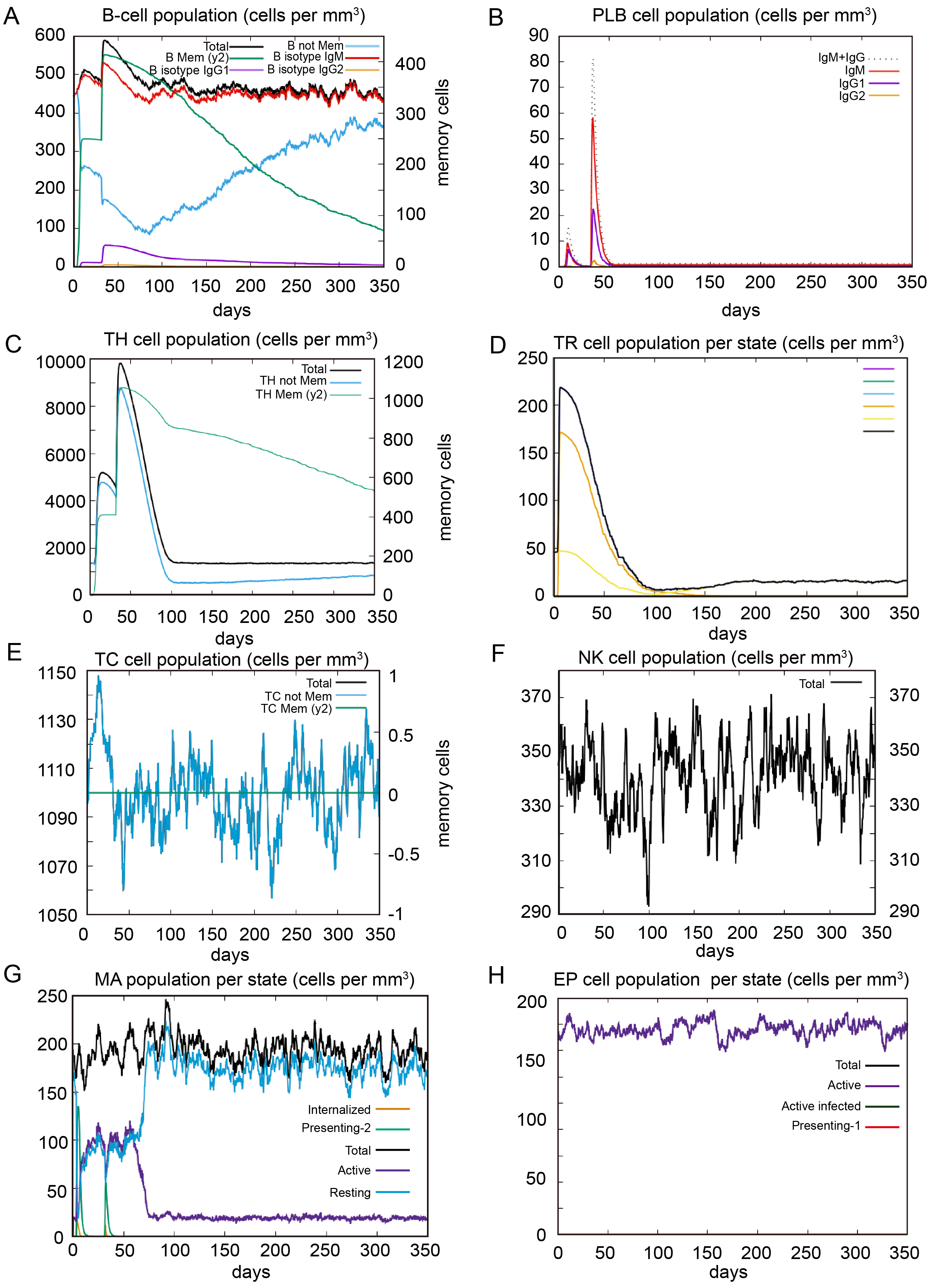
**

**Supplementary Fig. 1.** C-ImmSim presentation of immune simulation of the vaccine candidate MTBV8. Two injections were administered at 1^st^ and 29^th^ days, respectively (four weeks apart). (**A**) B lymphocyte count after the two injections; (**B**) Plasma B lymphocytes count after the two injections; (**C**) CD4^+^ T-helper (TH) lymphocyte count after the two injections; (**D**) Regulatory T (TR) lymphocyte count per entity state after the two injections; (**E**) ) CD8^+^ T-cytotoxic (TC) lymphocyte count after the two injections; (**F**) Natural killer (NK) cells count after the two injections (**G**) Macrophages count per entity state after the two injections; (**H**) Epithelial cells (EPC) count per entity state after the two injections.


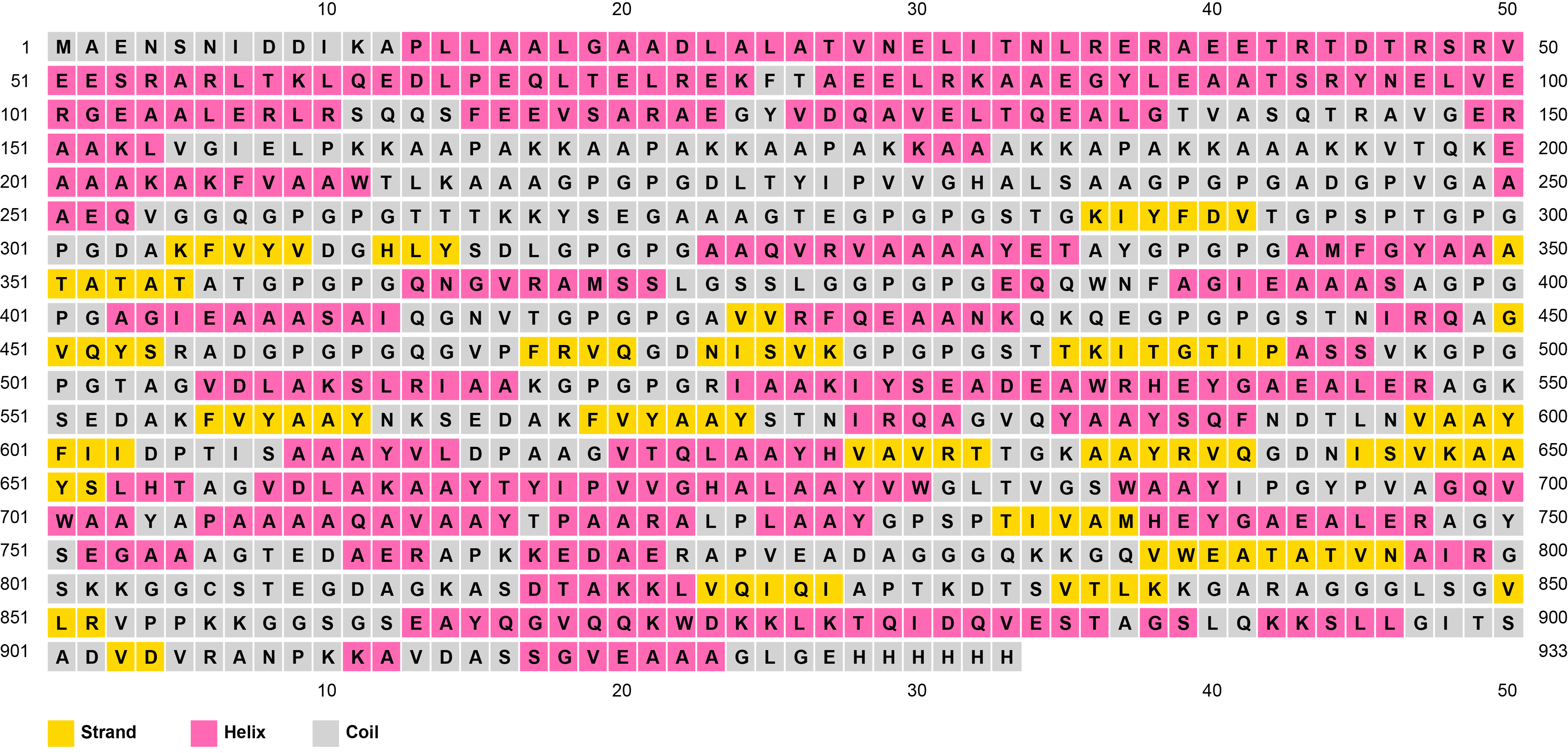


**Supplementary Fig. 2.** Secondary structure of the multi-epitope vaccine MTBV8. The chimeric protein consists of helices (42.12%), strands (11.79%), and coils (46.09%).


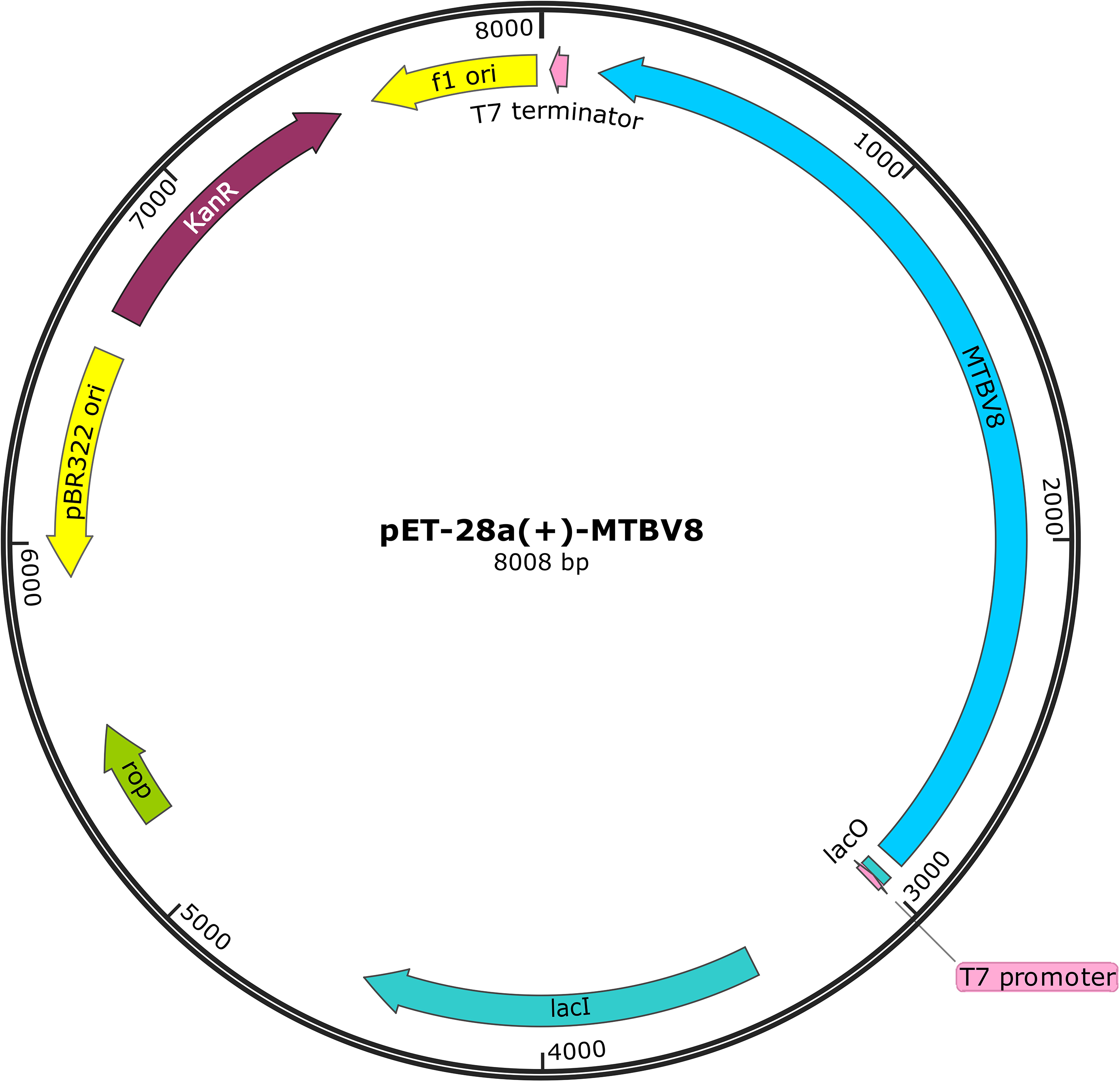


**Supplementary Fig. 3.** *In silico* cloning of *mtbv8* into the reverse strand of pET28a(+) vector. The DNA fragment of *mtbv8* was cloned into pET-28a(+) vector between the NcoI and XhoI restriction sites, generating pET-28a(+)-*mtbv8* recombinant plasmid (8008 bp).
